# Demographic history and population structure of *Anopheles pseudopunctipennis* in Argentina based on the mitochondrial *COI* gene

**DOI:** 10.1186/1756-3305-7-423

**Published:** 2014-09-04

**Authors:** María J Dantur Juri, Marta Moreno, Mónica J Prado Izaguirre, Juan C Navarro, Mario O Zaidenberg, Walter R Almirón, Guillermo L Claps, Jan E Conn

**Affiliations:** Instituto Superior de Entomología “Dr. Abraham Willink”, Facultad de Ciencias Naturales e Instituto Miguel Lillo, Universidad Nacional de Tucumán, Miguel Lillo 205, San Miguel de Tucumán, 4000 Tucumán, Argentina; Wadsworth Center, Griffin Laboratory, New York State Department of Health, Albany, NY 12159 USA; Department of Biomedical Sciences, School of Public Health, State University of New York-Albany, Albany, NY 12222 USA; Division of Infectious Diseases, School of Medicine, San Diego, George Palade Labs, School of Medicine, University of California, 9500 Gilman Drive, MC 0741, La Jolla, CA 92093 USA; Laboratorio de Biología de Vectores, Instituto de Zoología Tropical, Universidad Central de Venezuela, Av. Paseo Los Ilustres, Los Chaguaramos Apartado 48058, Caracas, 1041-A Venezuela; Coordinación Nacional de Control de Vectores, Ministerio de Salud de la Nación, Güemes 125, 4400 Salta, Argentina; Centro de Investigaciones Entomológicas de Córdoba, Facultad de Ciencias Físicas, Exactas y Naturales, Universidad Nacional de Córdoba, Av. Vélez Sarsfield 1611, 5016 Córdoba, Argentina

## Abstract

**Background:**

*Anopheles pseudopunctipennis* is an important malaria vector in the Neotropical region and the only species involved in *Plasmodium* transmission in the Andean foothills. Its wide geographical distribution in America, high preference for biting humans and capacity to rest inside dwellings after feeding, are attributes contributing to its vector status. Previous reports have tried to elucidate its taxonomic status, distinguishing populations from North, Central and South America. In the present study we used a mitochondrial marker to examine the demographic history of *An. pseudopunctipennis* in northwestern Argentina.

**Methods:**

Twelve localities were selected across 550 km of the distribution of this species in Argentina, including two near the Bolivian border and several in South Tucumán, for sampling. A fragment of the *cytochrome oxidase I* (*COI*) gene was sequenced and haplotype relationships were analyzed by a statistical parsimony network and a Neighbor-Joining (NJ) tree. Genetic differentiation was estimated with *F*_*S*T_. Historical demographic processes were evaluated using diversity measures, neutrality tests and mismatch distribution.

**Results:**

Forty-one haplotypes were identified, of which haplotype A was the most common and widely distributed. Neither the network nor the NJ tree showed any geographic differentiation between northern and southern populations. Haplotype diversities, Tajima’s *D*_*T*_ and Fu & Li’s *F* and *D* neutrality tests and mismatch distribution supported a scenario of Holocene demographic expansion.

**Conclusion:**

The demographic pattern suggests that *An. pseudopunctipennis* has undergone a single colonization process, and the ancestral haplotype is shared by specimens from all localities, indicating mitochondrial gene flow. Genetic differentiation was minimal, observed only between one northern and one southern locality. The estimated time of the population expansion of this species was during the Holocene. These data suggest that regional vector control measures would be equally effective in both northern and southern localities sampled, but also that insecticide resistant genes may spread rapidly within this region.

## Background

Malaria affects millions of people globally every year. In 2010, 216 million malaria cases were registered, 81% of them in Africa [1]. Approximately half of the world’s population lives in areas with some risk of malaria transmission, and in America this number is 137 million people [1, 2]. Differences in transmission intensity, the presence of several competent mosquito vector species, multiple parasite species, human migration and anthropogenic environmental changes, are some of the factors that contribute to wide variation in malaria [2]. In addition, the presence of a suitable vector with characteristics such as endophily, anthropophily, endophagy, longevity, high titer of sporozoites and high effective local vector population size are essential for transmission to occur [3].

In the Americas, different malaria vectors are associated with distinctive eco-regions [4]. Specifically *Anopheles* (*Anopheles*) *pseudopunctipennis* Theobald is involved in Andean foothills and coastal area malaria transmission [4]. The eco-regional classifications consider the anthropogenic environmental changes that can affect the distribution and abundance of the vector and, therefore, the intensity of *Plasmodium* transmission. For instance, the appearance of *An. pseudopunctipennis* on the dry coast of Peru was directly related to land use change from desert to irrigation for sugar cane and rice, creating suitable new species habitat [4].

*An. pseudopunctipennis* has an extensive distribution from the USA to northern Chile and northwestern Argentina, including the Caribbean Lesser Antilles, Trinidad and Tobago and Hispaniola Island [5–8]. Since the original description of this species by Theobald [9] until its redescription by Rueda *et al.* [10], several studies have attempted to evaluate its taxonomic status [11–15]. Estrada- Franco *et al.* [8, 13, 14] detected two different geographical populations (Mexican and South American) based on cross-mating experiments and fixed differences at two enzyme loci. Manguin *et al.* [15] using electrophoretic analyses, reported the presence of three *An. pseudopunctipennis* populations: one from the southern United States throughout Mexico and Guatemala; another extending from South America through Central America and Belize, both sharing an area of overlap in eastern Guatemala and southern Belize; and a third including only populations from the island of Grenada. Currently, *An. pseudopunctipennis* is considered to be a complex of at least 2 species [10]. However, there is not much information about demographic processes that could have lead, via allopatry, to speciation.

In Argentina, the historically wide geographical distribution of malaria appears to be reduced to the northwest, where it is still an important endemic parasitic disease [16–18]. Land use changes during the last century lead to a different level of malaria transmission that was indirectly linked to gradual changes in the yungas ecoregion, providing new breeding sites for the vector *An. pseudopunctipennis*. Until the 40’s, the northern area of the yungas was a preserved rainforest, whereas in the southern area cultivation included sugar cane, citrus and soybean crops. However, after the 40’s, the dynamics in the yungas were altered and the northern area began to exhibit major landscape modifications with severe forest exploitation, recurrent occurrence of fires and the increased pressure of farming [19] that required the presence of workers (a population naïve to malaria) in the area. These environmental alterations indirectly imply climatic changes, and, combined with human migration between southern Bolivia and northwestern Argentina since the second half of the 20^th^ century, could explain the current distribution and abundance of *An. pseudopunctipennis* mosquitoes and the regional malaria endemicity [18].

The use of the *cytochrome oxidase I* gene (*COI*) for population demographic analyses of Anophelinae species has been well-documented, i.e. Fairley *et al.* [20, 21] in *Anopheles* (*Nyssorhynchus*) *aquasalis* Curry and *Anopheles* (*Anopheles*) *punctipennis* Say, and Mirabello & Conn [22] and Pedro & Sallum [23] for *Anopheles* (*Nyssorhynchus*) *darlingi* Root, among others. The effects of geographical barriers and latitude can show not only differentiation among populations but also possible barriers to gene flow [21]. For instance, populations of *An. darlingi* from Central and South America appear to be separate from Amazonia and southern Brazil specimens, with the southern ones considered more ancestral [22], and barriers to gene flow were also detected along the Amazon River and in southern Brazil [23].

In the present study, we analyzed the demographic history and population structure of *An. pseudopunctipennis* from two areas of the yungas ecoregion of Argentina by *COI* to elucidate the history of this species, as a first attempt to compare populations throughout its range in the Americas.

## Methods

### Mosquito collection

**Figure 1:**
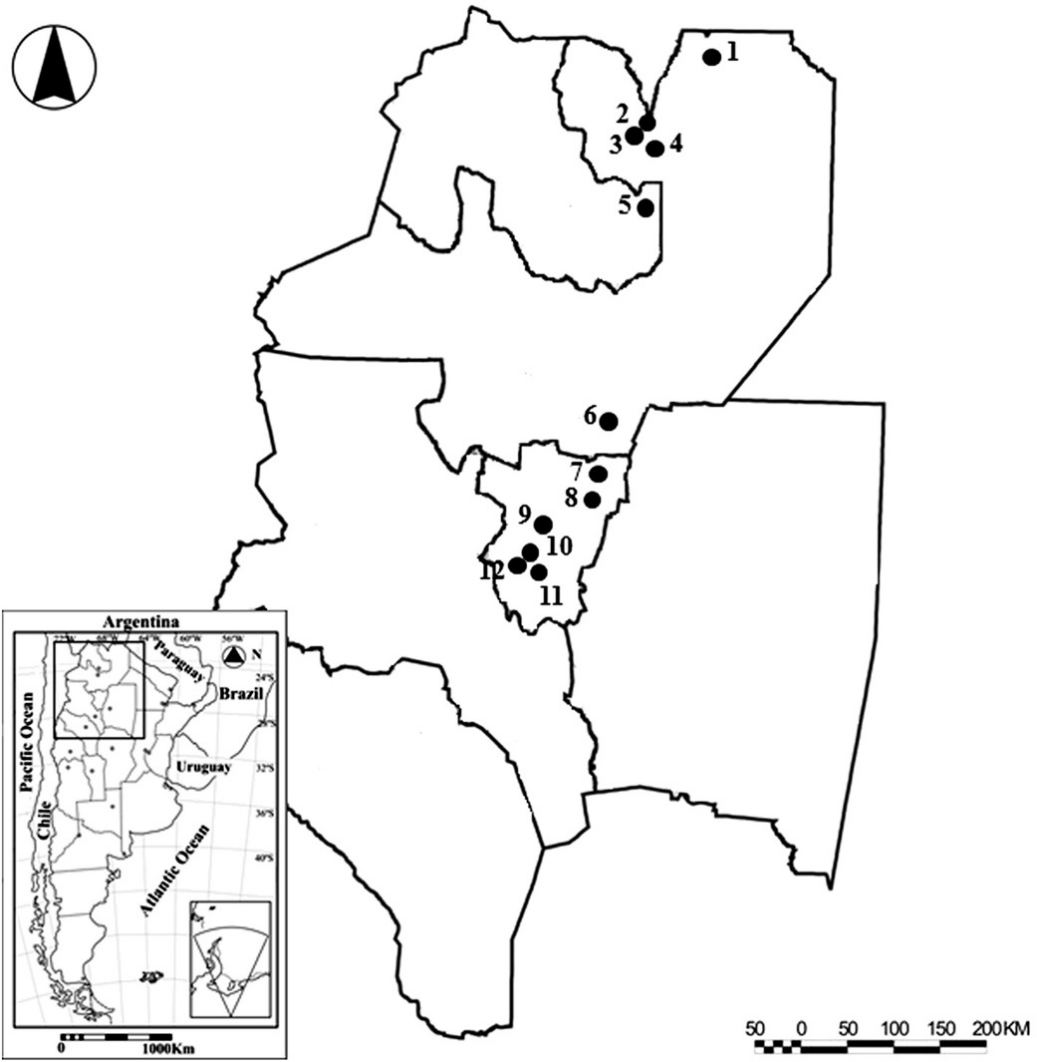
***Anopheles pseudopunctipennis*** **localities.** Collection localities of *An. pseudopunctipennis* in northwestern Argentina (yungas ecoregion): 1 = San Roquito-Tartagal, 2 = Aguas Blancas, 3 = El Oculto, 4 = San Ramón de la Nueva Orán, 5 = Finca Yuto, 6 = Rosario La Frontera, 7 = Vipos, 8 = El Cadillal, 9 = Potrero Las Tablas, 10 = Capitán Cáceres, 11 = La Florida, 12 = Sargento Moya (Tucumán Sur).

One hundred and sixty-five adult female *An. pseudopunctipennis* were collected from twelve localities in two areas, northern and southern yungas, situated in Salta, Jujuy and Tucumán provinces, northwestern Argentina (Figure 1, Table 1). These localities are included within the *An. pseudopunctipennis* geographical range reported by various authors [24–26]. The northern and southern yungas can be differentiated by vegetation and latitude [27, 28]. The northern area is characterized by anthropic activity, including timber harvesting and modification of land for agriculture use. However, some relicts of native vegetation represented by “palo blanco” and “palo amarillo” trees (*Calycophyllum multiflorum* Griseb. (Castelo) and *Phyllostylon rhamnoides* (Poiss.) Taubert), respectively, still remain. The southern area, namely the “tipa and pacará” forest, *Tipuana tipu* (Benth.) Kuntze and *Enterolobium contortisiliquum* (Vell.) Morong, respectively, has been modified by intensive sugar cane, soybean and citrus plantations, displacing more traditional and sustainable land use [27, 28].

**Table 1:** **Collection localities (name and geographical coordinates), number of** ***An. pseudopunctipennis*** **specimens (** ***N*** **) and haplotype occurrences and frequencies, in northwestern Argentina**

Sites	Localities	Latitude/longitude coordinates	***N***	Haplotypes	Collector/s
**North Yungas**					
1	San Roquito – Tartagal (SRT)	22°32′N, 63°49′W	20	A(9),B(1),B6(1),G(2),H(1),I(1),J(1), M(1),RR(1),V(1),Y(1)	MJDJ/EL/ACC
2	Aguas Blancas (AB)	22°43′N, 64°21′W	20	A(13),B(1),B3(1),B5(1),C(1),H(1), O(1),P(1)	MJDJ/EL/NV/JCH
3	El Oculto (EO)	23°06′N; 64°31′W	20	A(14),B1(1),D(1),Ñ(1),R(3)	MJDJ/EL/NV/JCH
4	San Ramón de la Nueva Orán (SRNO)	23°07′N, 64°19′W	19	A(15),B7(1),E(1),F(1),N(1)	MJDJ/EL/NV/JCH
5	Finca Yuto	23°63′N, 64°46′W	18	A(12),B(1),B2(1),H1(1),Q(1),Y(1)	MJDJ/EL/NV/JCH
**South Yungas**					
6	Rosario de la Frontera (RF)	25°48′N, 64°58′W	20	A(12),B(3),CH(1),LL(1),Q(1),RR(1),	MJDJ/GBG/CAVA
				S(1),Z1(1)	
7	Vipos (VP)	26°30′N, 65°21′W	17	A(15),B4(1)	MJDJ/GBG/CAVA
8	El Cadillal (EC)	26°36′N, 65°12′W	20	A(16),L(1),V(1),α(2)	MJDJ/GBG/CAVA
9	Potrero Las Tablas (PT)	26°54′N, 65°27′W	4	A(1),W(1),X(1),Z(1)	MJDJ/GBG/CAVA
**Tucumán Sur (TUCSUR)**				A(4),B8(1),K(1),T(1),U(1)	
10	Capitán Cáceres (CC)	27°10′N, 65°36′W	3		MJDJ, LO, GQ
11	La Florida (LF)	27°13′N, 65°33′W	2		MJDJ, LO, GQ
12	Sargento Moya (SM)	27°13′N, 65°39′W	2		MJDJ, LO, GQ

MJDJ: Maria Julia Dantur Juri, EL: Enrique Laci, NV: Neri Vianconi, JCH: Juan Carlos Hitzamatzu, GBG: Guillermina Begoña Galante, CAVA: Cecilia Adriana Veggiani Aybar, LO: Luis Oroño and GQ: Gabriela Quintana.

Collections were made by CDC light traps baited with carbon dioxide in 2005, 2007 and 2008 from 16:00 h-12:00 h in the yungas covering a latitudinal transect of ~550 km. Adult females were identified using the taxonomic key of Wilkerson & Strickman [29] and were deposited in the Instituto-Fundación Miguel Lillo Collection, Argentina (IMLA).

### DNA extraction and sequencing

DNA extractions were carried out from whole individual mosquitoes following the standard DNeasy Blood & Tissue Handbook protocol (Qiagen, CA, USA). A 1200 bp fragment of *COI* gene was amplified by polymerase chain reaction (PCR) using the UEA3 and UEA10 primer pairs [30]. Each PCR reaction was carried out using a Ready-To-Go-PCR Bead (GE Healthcare- Biosciences, NJ, USA) and performed on a PTC-200 thermal cycler (BioRad, Inc.). PCR products were purified with CentriSpin 40 columns (Princeton Separation, NJ, USA) and ExoSAP-IT (USB Corporation, Ohio, USA) and forward and reverse sequencing was performed at the Applied Genomic Technologies Core (Wadsworth Center, New York State Department of Health) on an ABI PRISM 3700 automated DNA sequencer.

Sequences for each individual sample were automatically aligned using Sequencher 3.0 (Gene Codes Corp, MI, USA) and corrected manually. The contig sequences were grouped together and aligned using MEGA version 3.1 [31]. In addition, the amino acid sequences were inferred to check for the presence of ambiguous stop codons that could suggest the presence of pseudogenes.

Few specimens were collected from the three most southern localities (10–12; Table 1) and the distances among them (7.45 – 9.9 km) are the lowest for all localities (range is 7.45 - 553.81 km). Therefore, these specimens were treated as a single population, TUCSUR (n = 7), for all analyses.

### Phylogenetic relatedness and demographic history

Statistical parsimony networks were constructed to assess relatedness among the *An. pseudopunctipennis COI* haplotypes using TCS 1.12 software [32] with a 95% connection limit. Genetic variation within populations was assessed by haplotype (*h*), sequence (*K*) and nucleotide diversity (*π*) indices using Arlequin 3.11 [33].

Statistical neutrality tests were performed to detect departures of DNA sequence variability from the expectations of the neutral theory of evolution [34]. Tajima’s *D* [35] is based on the difference between the estimates of the number of segregating sites and the average number of pairwise differences. The *D* and *F* tests proposed by Fu & Li [36] require data from intraspecific molecular polymorphism. Fu’s *F*_S_ test [37] is based on the haplotype frequency distribution and the *R*_*2*_ statistic [38] is based on the difference between the number of singletons per sequence and the average number of nucleotide differences. DnaSP v 5 [39] was used for all these calculations.

A mismatch analysis for each partition (north–south) and overall was carried out using Arlequin 3.11. The analysis compares the frequency distribution of pairwise differences between haplotypes with that expected under a model of population expansion [40–42]. To quantify the smoothness of the mismatch distribution, the raggedness (*r*) statistic was calculated and its significance was assessed using 10,000 replicates [43].

### Population genetic structure and gene flow

Genetic differentiation between populations of *An. pseudopunctipennis* was estimated by *F*_ST_ using Arlequin 3.11 [33]. The *F*_ST_ values were used as distance measures to create a NJ tree by DNAsp 4.50.3 [39]. Nei’s *G*_ST_ values were calculated to estimate population differentiation based on differences in allele frequencies and Nei’s *Nm*, the mean per generation estimate of the absolute number of migrants exchanged among populations [44]. The population structure was evaluated by analysis of molecular variance (AMOVA) in Arlequin 3.11 [33]. The hypothesis tested whether the northern and southern yungas represented distinct groups. A spatial analysis of molecular variance (SAMOVA) was performed, which combined genetic differentiation and geographical distance to define groups of geographically homogeneous populations and those with maximum differentiation from each other [45]. In addition, isolation by distance (IBD) was tested using a nonparametric Mantel with the web-based computer program IBDWS v.3.16 [46].

## Results

A fragment of 625-bp of the mitochondrial *COI* gene, from nucleotides 615–1269 was obtained. No stop codons were detected, indicating the mitochondrial origin of the DNA. All *COI* sequences are available at GenBank under accession numbers KC110039-KC110079. Forty-one haplotypes were identified, seven of which (A, B, H, Q, RR, V and Y) were shared between all northern and southern localities, accounting for 76.9% of the sequences. The dominant haplotype, A, represented 61.21% of the specimens, and was found in all localities (Table 1). Haplotype B was detected in four localities (SRT, AB, FY and RF) from the northern and southern areas. Unique haplotypes were distributed in all the localities.

Nucleotide and haplotype diversity values are depicted in Table 2. The highest haplotype diversity was in PT (1.0), followed by SRT (0.805). In general, the nucleotide diversity values were low and similar between populations and groups, slightly higher and more heterogeneous in the south but insignificantly so. Pairwise *F*_*ST*_ values were used to create the NJ tree. The *F*_*ST*_ values ranged from 0–0.177, and there was only one significant value, indicating a moderate differentiation (0.036) between the FY (Jujuy Province) and EC (Tucumán Province) localities, which are 66 km apart.

**Table 2:** **Haplotypes and nucleotide diversity values and segregating sites of** ***An. pseudopunctipennis*** **in northwestern Argentina**

Sites	Localities	***H***/***N***	***Hd***	***Pi***	***K***	***S***
	**North Yungas**					
1	San Roquito- Tartagal	11/20	0.805 (0.090)	0.00179 (0.00034)	1.173	9
2	Aguas Blancas	8/20	0.589 (0.130)	0.00163 (0.00056)	1.068	9
3	El Oculto	5/20	0.505 (0.126)	0.00102 (0.00033)	0.668	5
4	San Ramón de la Nueva Orán	5/19	0.385 (0.139)	0.00080 (0.00034)	0.526	5
5	Finca Yuto	7/18	0.568 (0.138)	0.00147 (0.00045)	0.960	7
	**Overall North**	28/97	0.579 (0.062)	0.00135 (0.00021)	0.887	27
	**South Yungas**					
6	Rosario La Frontera	7/20	0.636 (0.116)	0.00194 (0.00081)	1.268	11
7	Vipos	3/17	0.227 (0.129)	0.00054 (0.00034)	0.352	3
8	El Cadillal	4/20	0.363 (0.131)	0.00104 (0.00042)	0.678	5
9	Potrero Las Tablas	4/4	1.000 (0.177)	0.00229 (0.00056)	1.500	3
10	Tucumán sur	4/7	0.714 (0.181)	0.00262 (0.00117)	1.714	6
	**Overall South**	18/68	0.503 (0.076)	0.00142 (0.00035)	0.929	25
	**Total**	41/165	0.547 (0.048)	0.00138 (0.00019)	0.904	44

*H*: Number of *H*: Haplotypes, *N*: Number of sequences obtained, *Hd*: Haplotype diversity (Standar Deviation), *Pi*: Nucleotide diversity (Standar Deviation), *K*: Average number of nucleotide differences, *S*: Number of segregating sites. Tucuman sur includes the following localities: Capitán Caceres, La Florida and Sargento Moya.

Nei’s *G*_*ST*_ and *Nm* values detected low pairwise genetic differentiation and moderate gene flow between the north and south. The highest value (*G*_*ST*_ = 0.14210) was between Potrero Las Tablas (PT) and Vipos (VP), both in the south, separated by 50.5 km. Estimates of gene flow (*N*_*m*_) varied widely between populations, ranging from 1.51 to 614.50; the lowest was between Potrero Las Tablas (PT) and Vipos (VP) (*N*_m_ ~ 1.51). Negative values were related to small population sizes and lack of gene flow [44, 47]. In our study, PT is represented by only four individuals, which might be providing a false inference and misinterpretation of results.

**Figure 2:**
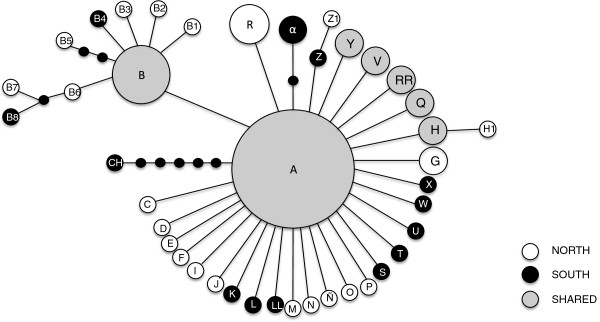
**Statistical parsimony network of 41 haplotypes of** ***An. pseudopunctipennis.*** Letters represent the haplotypes observed in twelve localities shown in Table 1. Single mutational events are indicated by lines, and missing or undersampled haplotypes by filled black ovals.

AMOVA findings were consistent with the apparent lack of structure within *An. pseudopunctipennis* in the yungas. Variation among the northern and southern groups was almost negligible (*Φ*_SC_ = -0.00429). The populations differed significantly from each other (*Φ*_CT_ = 0.442) and were responsible for 97.4% of the variance, although the value was non-significant. The remaining difference (3.54%) was explained by non-significant differentiation of haplotypes within populations (*Φ*_ST_ = 0.016). SAMOVA did not show any geographical limit within populations, and *k* = 3 was the partition providing the highest significant *F*_CT_ (0.27), although it did not separate the northern-southern populations. Instead, TUCSUR (southern) and RF (northern) group separately from the other localities (grouping together). The Mantel test was not significant (*R* = 0.0181, *p* = 0.385) showing that there was no geographic association with the genetic differentiation.The statistical parsimony network revealed a star-like topology (Figure 2). A was the most abundant haplotype and the only one found in all localities, and was designated as ancestral. The second most common haplotype (B) was distributed in the north and the south. Twenty-seven haplotypes were connected to the ancestral haplotype by one mutational step and 31 haplotypes were represented by a single individual. The maximum number of mutational steps observed in the network was six, between haplotypes A (found in all localities) and CH (restricted to locality 6, Rosario La Frontera).

Tajima’s *D* and Fu & Li’s *F* and *D* neutrality tests showed significant negative values in the northern and southern populations (Table 3) and there were also indications of a recent population expansion for the complete data set. On the other hand, Fu’s *F*_*S*_ test estimated negative values in both areas and overall, although none were significant.

**Table 3:** **Indices and statistics used to measure** ***An. pseudopunctipennis*** **polymorphisms**

Sites	Localities	***H/N***	***D***	D*	F*	***Fu’s***	***R*** ^***2***^
	**North Yungas**						
1	San Roquito- Tartagal	11/20	-2.04083*	-2.39168	-2.65401*	-9.594	0.0583
2	Aguas Blancas	8/20	-1.99058*	-2.75174*	-2.93379*	-4.721	0.0830
3	El Oculto	5/20	-1.60863	-2.01240	-2.19139	-2.175	0.1055
4	San Ramón de la Nueva Orán	5/19	-1.96578*	-2.75581*	-2.92267*	-2.913	0.1094
5	Finca Yuto	7/18	-1.79140	-2.33361	-2.51578	-3.955	0.0851
	**Overall North**	28/97	-2.56303***	-5.84694**	-5.47713**	-41.203	0.0205
	**South Yungas**						
6	Rosario La Frontera	7/20	-2.09572*	-2.96623**	-3.14728**	-2.682	0.1209
7	Vipos	3/17	-1.70573	-2.25481	-2.41419	-0.963	0.1709
8	El Cadillal	4/20	-1.58577	-1.21271	-1.51924	-0.882	0.1026
9	Potrero Las Tablas	4/4	-0.75445	-0.75445	-0.67466	-2.367	0.1443
10	Tucumán sur	4/7	-1.52412	-1.60880	-1.73234	-0.428	0.2259
	**Overall South**	18/68	-2.57256***	-5.36858**	-5.17929**	-18.985	0.0344
	**Total**	41/165	-2.66941***	-6.84292**	-6.08110**	-70.455	0.0153

*H*: Number of Haplotypes, *N*: Number of sequences obtained, *D*: Tajima’s Statistic, D*: Fu and Li D* Statistic, F*: Fu and Li F* Statistic, *Fu’s*: Fu’s Fs Statistic, *R* ^*2*^: Ramos-Onsins & Rozas, p < 0.05, **p < 0.02, ***p < 0.001.

Tucumán sur includes the following localities: Capitán Caceres, La Florida and Sargento Moya.

**Figure 3:**
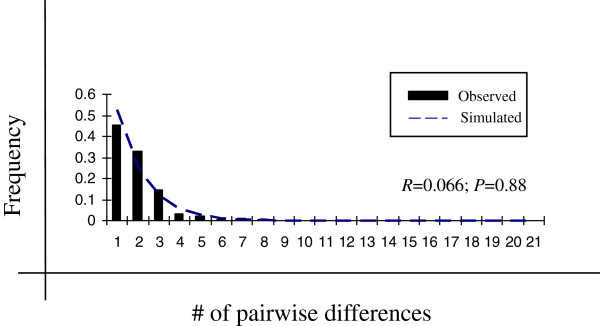
**Observed and simulated mismatch frequency distributions under population expansion model for** ***Anopheles pseudopunctipennis*** **.**

The mismatch distribution for the complete data set is presented in Figure 3. The population expansion model was not rejected in either case (*p* = 0.058, 0.126 and 0.088, respectively), which was consistent with a model of sudden expansion for each partition.

To calculate the time of population expansion, *t* = τ/2 μ was used, where μ is the mutation rate per site per generation [22, 40]. The mutation rate in *Drosophila* was used: 10^-8^/site/year [48] and 10 generations/year [49]. The analysis was done for all the samples, since there were no evidence for different populations. The estimate of τ was 0.924 giving an expansion estimated to 7, 392 years ago (95% CI, 32–15, 872), in the Holocene.

## Discussion

The present study shows very little genetic structure of the malaria vector *Anopheles pseudopunctipennis* in the yungas ecoregion of Argentina based on one mitochondrial marker. Therefore, the hypothesis of genetic differentiation or restriction of gene flow between the northern and southern yungas areas of Argentina is rejected, and there appears to be a single metapopulation in this region of northwestern Argentina. The AMOVA analysis did not reveal significant genetic differentiation when populations were grouped by distinct ecotype, the north and south divisions. Similarly, no boundaries were defined by SAMOVA for the complete data set. Previous findings in *An. pseudopunctipennis* in North, Central and South America showed deep population structure within this group using other molecular markers [8, 13–15]. Our results suggest that *An. pseudopunctipennis* from the yungas is a unique population with *COI* mitochondrial gene flow among localities with similar demographic history. However, to test whether the current degradation of the natural ecosystem and landscape change is affecting the population further studies focused on markers with a faster mutation rate would be desirable [8, 13–15].

When compared to other neotropical malaria vectors, the genetic differentiation between two populations of the malaria vector *An. albimanus* from Colombia showed similar results [50]; in spite of the deep population structure of the species, low genetic differentiation was observed between Caribbean and Pacific populations that correspond to different biogeographical regions. Loaiza *et al.* [51] studying *An. albimanus* populations from southern Central America founded significant genetic structure between populations from Costa Rica and western Panama compared with those from central-eastern Panama, whereas in our study, divergence within groups was shallow and statistically insignificant.

In addition, the low nucleotide diversity and the absence of isolation by distance observed for *An. pseudopunctipennis* is similar to results obtained for *An. darlingi* and *An. albimanus* by Mirabello & Conn [22], by De Merida *et al*. [52] and by Molina Cruz *et al*. [53], linking this to small effective migration rates and effective population size and /or genetic drift. The fact that in *An. pseudopunctipennis* nucleotide diversity was low, but not haplotype diversity, can be explained by rapid population growth from ancestral populations.

The N*m* values between northern and southern populations showed not only high gene flow between them (N*m* > 1) but also negative values (N*m* <1) related to low gene flow, where significant population differentiation could occur through genetic drift. The more ancestral and diverse haplotypes of *An. pseudopunctipennis* were observed in both the north and south. As reported by Mirabello & Conn [22], Molina-Cruz *et al.* [53] and Kambhampati & Rai [54], older populations have a higher diversity; this seems to be the case for San Roquito-Tartagal (northern area) and Potrero Las Tablas and Tucumán Sur (southern area). On the basis of the presence of shared haplotypes between northern and southern populations, *An. pseudopunctipennis* has undergone an extensive expansion population process. In fact, the presence of a dominant haplotype represents an ancestral lineage, because older haplotypes have had more time to spread, leading to a higher frequency and geographic distribution [55]. Furthermore, mismatch distribution for the entire group exhibited a unimodal pattern, suggesting that demographic expansions occurred. Although Pleistocene population expansion has been detected in other Neotropical anophelines, such as *An. darlingi* (Amazonian region of Brazil) [22, 56], *Anopheles marajoara* showed a more recent population expansion in Brazil, when it was compared to *An. darlingi* [57]. This difference may reflect the contraction and re-expansion cycles of Amazonian savanna, which created differences in the availability of habitats for breeding of these two species [58, 59]. Unfortunately, few data from southern Argentina during the Holocene are available, although the Andean glaciations are correlated with the amount of available moisture more than a fall in temperature [59]. Immature stages of *An. pseudopunctipennis* are commonly found in riverside pools colonized by filamentous algae of the genus *Spirogyra* in the foothills of the mountainous Mesoamerica region [60–62]. Therefore, one hypothesis that could be tested is that climatic oscillations during the Pleistocene together with modifications in vegetation could favor the presence of breeding sites for this species during the Holocene.

The development of alternative breeding sites, or the increase of new suitable breeding sites because of the anthropic alterations of natural habitats, such as development of crops [60], may be a concern due to the possibility of colonization of new areas where the vector (or new vectors) was absent before. Thus, malaria vector surveillance should be included in the strategies of disease control in the area.

## Conclusion

This is the first report of the use of the mitochondrial *COI* gene to study the population demography of *An. pseudopunctipennis* in America, although the study was restricted to the Argentinian distribution. Results do not support the existence of northern and southern *An. pseudopunctipennis* population differentiation. Instead, this metapopulation seems to have undergone a single colonization process, without differentiation between northern and southern localities, with fairly high gene flow among them and evidence of a Holocene expansion.

In summary, this research reports the pattern of the genetic variability and gene flow among northwestern Argentinian localities of *An. pseudopunctipennis*. This study provides important baseline data that suggest that similar vector control measures should work in both the north and south, and also, if insecticide resistance evolves, it would likely spread fairly rapidly in this area.
